# Prevalence of Anemia among Adults with Newly Diagnosed HIV/AIDS in China

**DOI:** 10.1371/journal.pone.0073807

**Published:** 2013-09-18

**Authors:** Yinzhong Shen, Zhenyan Wang, Hongzhou Lu, Jiangrong Wang, Jun Chen, Li Liu, Renfang Zhang, Yufang Zheng

**Affiliations:** Department of Infectious Diseases, Shanghai Public Health Clinical Center, Fudan University, Shanghai, China; Alberta Provincial Laboratory for Public Health/University of Alberta, Canada

## Abstract

**Background:**

The prevalence of anemia among antiretroviral-naïve HIV-infected patients in China has not been well characterized. We conducted a cross-sectional study to estimate the prevalence of anemia among Chinese adults with newly diagnosed HIV/AIDS.

**Methods:**

One thousand nine hundred and forty-eight newly diagnosed HIV-infected patients in China were selected during 2009 and 2010. Serum samples obtained from each individual were collected to measure hemoglobin levels. Demographics and medical histories were recorded. Factors associated with the presence of anemia were analysed by logistic regression.

**Results:**

Among the 1948 patients, 75.8% were male. Median age was 40 years (range: 18–80 years). The overall prevalence of anemia among HIV-infected patients was 51.9% (51.5% among men, 53.2% among women). The prevalences of mild anemia, of moderate anemia, of severe anemia were 32.4%, 17.0%, and 2.5%, respectively. The prevalence of anemia was higher among ethnic minority patients than among the Han patients (70.9% versus 45.9%). The prevalence of anemia increased with increasing age (49.6%, 53.5% and 60.1% among patients who were 18–39, 40–59, and ≥60 years of age respectively) and with decreasing CD4 count (14.0%, 22.4%, 50.7%, and 74.6% among patients with CD4 count of ≥350, 200–349, 50–199, and <50 cells/mm^3^ respectively). The logistic regression analysis showed that older age, lower CD4 count and minority ethnicity were significantly associated with an increased risk of anemia.

**Conclusions:**

Anemia is highly prevalent among Chinese adults with newly diagnosed HIV/AIDS, but severe anemia is less prevalent in this population. Older age, lower CD4 count and minority ethnicity are associated with an increased risk of anemia.

## Introduction

Hematologic abnormalities are among the most common manifestations of advanced HIV infection and AIDS [1]–[3]. Of these abnormalities, anemia is the most common hematologic manifestation, affecting 60% to 80% of HIV-infected patients in late-stage disease [1], [4], [5]. Anemia also comprises a significant proportion of the adverse events secondary to antiretroviral therapy (ART) [6], [7]. Hematologic abnormalities have been documented as strong independent predictors of morbidity and mortality in HIV-infected patients [8]. A large epidemiological study of 32,867 HIV-infected adults and adolescents in USA found that the risk of death was 170% greater for persons with persistent anemia compared with those whose anemia had resolved [9]. This study showed that anemia, particularly anemia that does not resolve, is associated with shorter survival of HIV-infected patients [9]. Anemia is also one of the strongest predictors of HIV mortality and poor responses to ART [8]. Reduction of anemia is thus one of the key components of medical care for HIV-infected patients.

In different study settings, the prevalence of anemia in persons with AIDS has been estimated at 63% to 95%, making it more common than thrombocytopenia or leukopenia in patients with AIDS [9]. Although anemia is the most common hematologic manifestation of HIV infection, and may have considerable impact on patients’ well-being, treatment and care, so far the prevalence of anemia among newly diagnosed HIV-infected patients in China has not been extensively studied. Such information for newly diagnosed HIV-infected patients may help to inform treatment of HIV-infected individuals. Accordingly, the purpose of the present study was to estimate the prevalence of anemia among Chinese adults with newly diagnosed HIV/AIDS, and to identify demographic and HIV-related factors that were associated with the presence of anemia.

## Methods

### Ethics Statement

Written informed consent was obtained for all subjects; consent forms and procedures, as well as survey protocol, were approved by the Shanghai Public Health Clinical Center Ethics Committee. No patient identifiers were included in the dataset used for this analysis.

### Study Population

We conducted a cross-sectional survey on HIV/AIDS in China’s HIV epidemic provinces and municipalities including Xinjiang, Jiangxi, Henan, Heilongjiang, Guangdong, Shaanxi, Guangxi, Hunan, Shanghai and Yunnan during 2009 and 2010. The survey subjects were newly diagnosed HIV-infected patients who had not received ART. Subjects aged 18 years or more at the time of enrolment with documented HIV infection were eligible for this study. Patients on ART were excluded from the study. All patients were confirmed to be positive for HIV antibody through laboratory detection, and the diagnosis was in line with national HIV/AIDS diagnostic criteria.

### Blood Samples

For newly diagnosed HIV-infected patients, three-milliliter venous blood samples were obtained from each individual for the measurement of hemoglobin levels. Hemoglobin level was determined in each blood sample with a CELL DYN 3200 hematology analyzer (Abbott Laboratories, USA), at the clinical laboratories in each province. All the study laboratories successfully completed a standardization and certification program.

### Study-Outcome Definitions

Results of hemoglobin level testing were categorized as follows: anemia [(hemoglobin level<120 g/l (men) or 110 g/l (women )], mild anemia [(hemoglobin level>90 g/l and <120 g/l (men) or 110 g/l (women )], moderate anemia (hemoglobin level>60 g/l and ≤90 g/l), and severe anemia (hemoglobin level≤60 g/l). Anemia was defined as either mild anemia, moderate anemia, or severe anemia.

### Data Collection

Data were collected according to standardised criteria. On enrolment, standardised data collection forms were completed at the sites providing information from patients interview and patient case notes. Data collected on newly identified cases included demographic information, risk-behavior information (injection drug use, history of heterosexual or homosexual sex, receipt of blood transfusion), and laboratory test results. Variables of interest included age, sex, HIV transmission route, and CD4 count. Age was denoted as <40, 40–59, or ≥60 years. HIV transmission route was categorized as sexual contact (including homosexual or heterosexual), blood (including blood transfusion or injection drug use), or unknown transmission risk. CD4 count was denoted as <50, 50–199, 200–349 or ≥350 cells/mm^3^.

### Statistical Analysis

SPSS software for Windows (version 11.5) was used for statistical analysis. Continuous variables were computed with standard methods and are presented as mean and standard deviations (SD). Categorical variables are reported as frequencies and percentage of each category. We performed one-way ANOVA to compare hemoglobin levels among patients with different CD4 count. Correlations between hemoglobin level and CD4 count were evaluated using the Pearson’s correlation test. A chi-square test was applied for categorical attributes. The odds ratio and 95% confidence intervals were calculated to assess the relationship between each risk factor and the risk of anemia; to adjust for the effects of potential confounders, we used a logistic regression model. All variables included in the models were determined a priori based on epidemiological importance and biological plausibility. Variables included in the models were age, sex, ethnicity, CD4 count, and HIV transmission route. The statistical test was two-tailed and performed at a level of statistical significance of 0.05.

## Results

### Patient Characteristics

We included a total of 1948 adults with newly diagnosed HIV/AIDS. Table 1 describes the basic characteristics of the study population. The study sample was primarily male (75.8%) (1476), the mean age was 40 years (41 years for males, 38 years for females), 24.1% (470) were ethnic minorities, and the mean CD4 count was 136 cells/mm^3^. Most patients acquired HIV through sexual contact (74.2%) (1446).

**Table 1:** Basic characteristics of 1948 newly diagnosed HIV/AIDS patients in China.

Characteristic	Patient No. (%)
Age, years	
18–39	1082 (55.5)
40–59	708 (36.3)
≥60	158 (8.2)
Mean age (range)	40 (18, 80)
Sex	
Male	1476 (75.8)
Female	472 (24.2)
HIV transmission category	
Sexual contact	1446 (74.2)
Blood	340 (17.5)
Unknown transmission risk	162 (8.3)
CD4 count, cells/mm^3^	
<50	796 (40.9)
50–199	611 (31.4)
200–349	370 (19.0)
≥350	171 (8.7)
Mean CD4 count (range)	136 (1, 891)
Ethnicity	
Han	1478 (75.9)
Other (minority)	470 (24.1)
Anemia	
Overall	1011 (51.9)
Mild	631(32.4)
Moderate	332(17.0)
Severe	48(2.5)

### Hemoglobin Levels among Men and Women with HIV/AIDS

**Figure 1:**
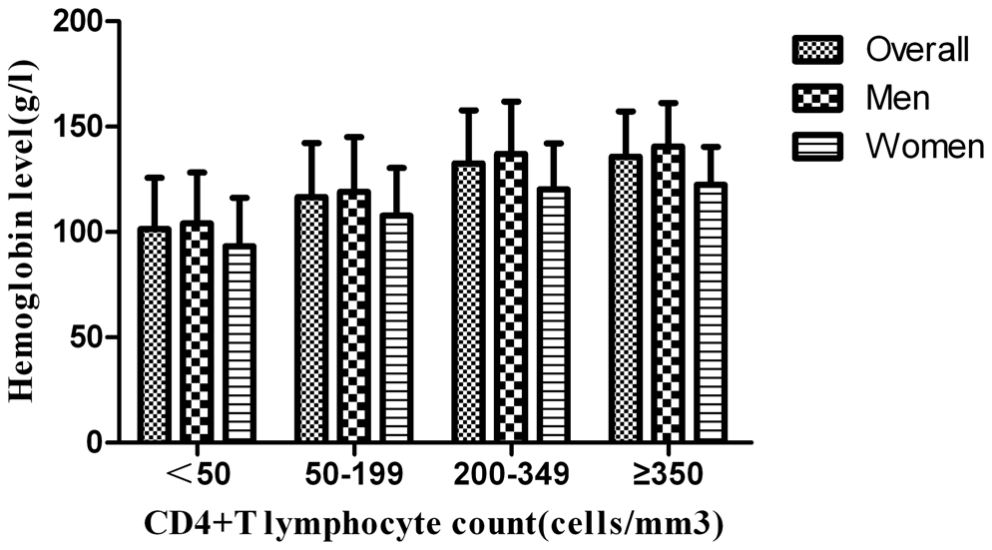
The mean hemoglobin level among men and women according to CD4 count. The numbers of men with CD4 counts of <50, 50–199, 200–349, and ≥350 cells/mm^3^ were 604, 474, 272, and 126, respectively. The numbers of women with CD4 counts of <50, 50–199, 200–349, and ≥350 cells/mm^3^ were 192, 137, 98, and 45, respectively. One-way ANOVA was used to compare hemoglobin levels among patients with different CD4 count. The mean hemoglobin level showed an increasing trend with increasing CD4 count (*P*<0.001 overall, men, and women).

The mean hemoglobin level was 118.1±28.2 g/l among men and 105.8±25.1 g/l among women, respectively. Figure 1 describes the mean hemoglobin level among men and women according to CD4 count. The mean hemoglobin level showed an increasing trend with increasing CD4 count (*P*<0.001 overall, men, and women). Using the Pearson’s correlation, there was a significant and positive correlation (r^2^ = 0.2079; *P*<0.001) between hemoglobin level and CD4 count.

### Prevalence of Anemia among Men and Women with HIV/AIDS

Among the 1948 patients, 1011 (51.9%) had anemia (Table 1). Among 1476 male patients, 760 (51.5%) had anemia; among 472 female patients, 251 (53.2%) had anemia. The prevalence of anemia did not differ significantly according to sex (*P* = 0.523). The prevalences of mild anemia, of moderate anemia, of severe anemia were 32.4%, 17.0%, and 2.5%, respectively (Table 1). The prevalences of mild anemia, of moderate anemia, of severe anemia were 33.9%, 15.7% and 1.9% among men and 27.5%, 21.4% and 4.2% among women, respectively. The prevalences of moderate anemia and of severe anemia among women were significantly higher than those among men (*P* = 0.004, *P* = 0.004), the prevalence of mild anemia was significantly higher among men than among women (*P* = 0.01).

### Prevalence of Anemia in Patients with Different CD4 Count

**Figure 2:**
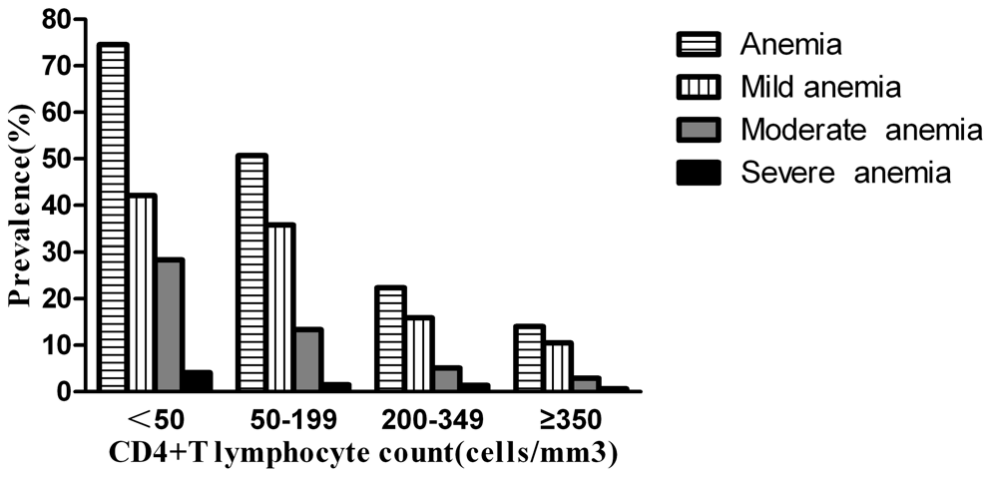
Prevalence of anemia, mild anemia, moderate anemia, and severe anemia among patients with different CD4 count. The numbers of patients with CD4 counts of <50, 50–199, 200–349, and ≥350 cells/mm^3^ were 796, 611, 370, and 171, respectively. A chi-square test was applied to compare differences in prevalence of anemia, mild anemia, moderate anemia, and severe anemia among patients with different CD4 count. The prevalence of anemia, of mild anemia, of moderate anemia, of severe anemia increased with decreasing CD4 count (*P*<0.001, *P*<0.001, *P*<0.001, *P* = 0.001).

Figure 2 describes the prevalences of anemia, mild anemia, of moderate anemia, of severe anemia among patients with different CD4 counts. The overall prevalence of anemia was 14.0%, 22.4%, 50.7%, and 74.6% among patients with CD4 counts of ≥350, 200–349, 50–199, and <50 cells/mm^3^, respectively. The overall prevalence of anemia increased with decreasing CD4 count (*P*<0.001). The prevalence of mild anemia, of moderate anemia, of severe anemia increased with decreasing CD4 count (*P*<0.001, *P*<0.001, *P* = 0.001).

### Prevalence of Anemia in Patients with Different Age

**Figure 3:**
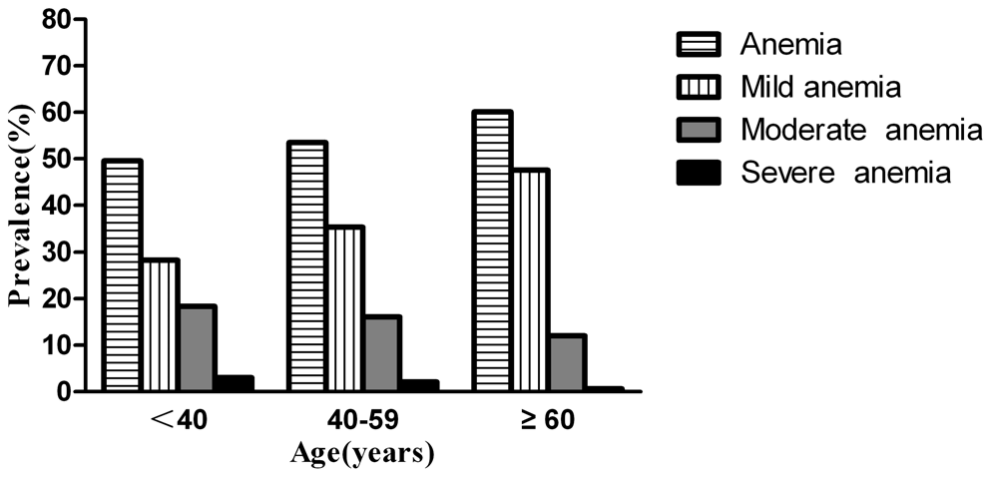
Prevalence of anemia, mild anemia, moderate anemia, and severe anemia among patients with different age. The numbers of patients who were 18–39, 40–59, and ≥60 years of age were 1082, 708, and 158, respectively. A chi-square test was applied to compare differences in prevalence of anemia, mild anemia, moderate anemia, and severe anemia among patients with different age. The prevalence of anemia increased with increasing age (*P* = 0.026). The prevalence of mild anemia increased with increasing age (*P*<0.001). The prevalences of moderate anemia and of severe anemia did not differ significantly according to age (*P* = 0.098, *P* = 0.154).

Figure 3 describes the prevalences of anemia, mild anemia, moderate anemia, and severe anemia among patients according to age. The prevalence of anemia was 49.6%, 53.5% and 60.1% among patients who were 18–39, 40–59, and ≥60 years of age, respectively. The prevalence of anemia increased with increasing age (*P* = 0.026). The prevalence of mild anemia increased with increasing age (*P*<0.001), the prevalences of moderate anemia and of severe anemia did not differ significantly according to age (*P* = 0.098, *P* = 0.154).

### Prevalence of Anemia in Patients according to Ethnicity

The prevalence of anemia was 45.9% and 70.9% among the Han patients and ethnic minority patients, respectively. The prevalence of anemia among ethnic minority patients was higher than that among the Han patients (*P*<0.001). The prevalences of mild anemia, of moderate anemia, of severe anemia were 29.5%, 14.5% and 1.8% among the Han patients and 41.5%, 24.9% and 4.5% among ethnic minority patients, respectively. The prevalences of mild anemia, moderate anemia and of severe anemia among ethnic minority patients were significantly higher than those among the Han patients (*P*<0.001, *P*<0.001, *P* = 0.001).

### Risk Factors for Anemia among Newly Diagnosed HIV-Infected Adults

In a multivariate analysis using a logistic regression model, we analyzed factors associated with the presence of anemia. Table 2 summarizes the results of the final regression model. Older age, lower CD4 count and minority ethnicity were significantly associated with an increased risk of anemia. HIV transmission route and sex failed to show an association with the presence of anemia.

**Table 2:** Identification of risk factors for the presence of anemia, results of the regression model.

Risk factor	P value	Odds ratio	95% Confidence interval
Age, per 20-year increment	0.025	1.202	(1.023, 1.411)
Minority ethnicity	<0.001	2.695	(2.099, 3.460)
CD4 count, per decrease of 150 cells/mm^3^	<0.001	2.919	(2.585, 3.297)
Sex	0.075	1.243	(0.979, 1.580)
HIV transmission route			
Sex contact	0.070	1.000	–
Blood	0.057	1.305	(0.993, 1.715)
Unknown transmission risk	0.126	1.331	(0.923, 1.920)

## Discussion

In this study, we observed a high prevalence of anemia among Chinese adults with newly diagnosed HIV/AIDS. The subjects were of broad geographic origin, which suggests that anemia is relatively prevalent among newly diagnosed HIV-infected patients in China. This constitutes further evidence of the need for monitoring hematologic parameters of HIV-infected patients, both before ART initiation and routinely during treatment, to ensure that mortality and morbidity are minimized and quality of life optimized. Routine blood tests are currently recommended for HIV-infected patients both before and after initiating HIV treatment by HIV care and treatment guidelines. In our study, the overall prevalence of anemia among newly diagnosed HIV-infected patients was 51.9%, with the majority of patients having mild to moderate anemia. Thus severe anemia appears to be less common in the study population.

There may be important biological implications to our finding that the mean hemoglobin level increased with increasing CD4 count, and that lower CD4 count was associated with an increased risk of anemia, in other words that the prevalence and severity of anemia increased with decreasing CD4 count. This suggests that the presence of anemia among newly diagnosed HIV-infected patients is related to HIV infection.

Anemia in HIV-infected patients is likely to be multifactorial [9]. Our study population was newly diagnosed HIV-infected patients who had not received ART, hence anemia among these patients was not caused by antiretroviral drugs. The pathophysiology of HIV-associated hematologic abnormalities may involve direct HIV infection of bone marrow progenitors, abnormal regulation of hematopoiesis and/or autoimmune phenomena [1]. Direct infection of marrow precursor cells has been hypothesized, but not proven [10]. HIV infection alone, without other complicating illnesses, may produce anemia in some patients [11]. The pathophysiology of HIV-associated anemia may involve three basic mechanisms: decreased red blood cell (RBC) production, increased RBC destruction, and ineffective RBC production [4]. Therefore, it is important to identify anemic patients and to consider HIV as a possible underlying cause. At the same time patients with HIV infection should be investigated and treated for anemia to reduce the morbidity of the patient.

Our findings are consistent with several published studies, which also indicate high anemia prevalence and similar associated risk factors in HIV-infected patients. Meidani and colleagues conducted a cross-sectional study [4] to investigate the prevalence of anemia and its related factors in HIV positive patients in Isfahan, Iran. In that study, the overall prevalence of anemia was 71%, with the majority of patients having mild to moderate anemia. Mild to moderate anemia and severe anemia occurred in 67% and 4% of patients, respectively. Mildvan *et al*. found that the prevalence of anemia in HIV-infected patients receiving no ART was 39.7%, and that anemia was more prevalent among patients with CD4 count <200 cells/mm^3^ [7]. In a cross-sectional study in the Mexican patients without ART, Mata-Marin and colleagues found a positive correlation between hemoglobin and CD4 count. That study also showed that CD4 count<200 cells/mm^3^ was associated with an increased risk of anemia [12]. Another study showed that the prevalence of anemia was 69.17% among ART naive patients in Benin City, Nigeria [13]. Parinitha *et al*. found that the prevalence of anemia was 84% among ART naive patients in India [14]. That study revealed a significant increase in the number of cases of anemia with decreasing CD4 counts. Based on the results of our study and in accordance with the literature on this topic, we conclude that anemia is highly prevalent among newly diagnosed HIV-infected patients, and it is more frequent with progression of HIV disease. The reported prevalence of anemia in HIV-infected populations differed significantly, with differences in prevalence explained by differences in the demographic characteristics and criteria of anemia used.

Our study showed that anemia was more frequent among ethnic minority patients than among the Han patients. Minority ethnicity was significantly associated with an increased risk of anemia, increasing the odds by a factor of 2.695. This association could be because of environmental and nutritional factors, socio-economic status and lifestyle or a combination of these factors. It is also possible that minority ethnicity is a marker for other factors that are associated with increased incidence of anemia but that were not included in our analysis. Mildvan *et al*. found that anemia in HIV-infected patients receiving no ART was more prevalent among blacks [7]. A study [9] from USA showed that the incidence of anemia is associated with black race. These findings suggest that race/ethnicity can be a factor associated with the presence of anemia among HIV-infected patients.

In this study population, we found that moderate to severe anemia was more common in women than in men, and that mild anemia was more common in men than in women. Mildvan *et al*. found that anemia in HIV-infected patients receiving no ART was more prevalent among men, and that marked anemia was more common in women [7]. Another two studies [15], [16] showed that female gender was significantly associated with anemia among HIV-infected individuals. Thus our findings are not inconsistent with gender influencing the frequency and severity of anemia among HIV-infected patients. However, we did not find a significant association between gender and the presence of anemia among newly diagnosed HIV-infected patients by a logistic regression model; the overall prevalence of anemia in our study population did not differ significantly according to gender. Therefore, the possible association between gender and anemia among newly diagnosed HIV-infected persons still needs further study.

In agreement with other reports [17], age is a factors influencing the prevalence of anemia in HIV-infected patients. Our study demonstrated that prevalence of anemia increased with increasing age. Older age was significantly associated with an increased risk of anemia. Injection-drug use may also influence the prevalence of anemia in HIV-infected patients [17]. However, HIV transmission route failed to show association with the presence of anemia in our study.

Some studies [18]–[20] showed that ART was associated with an improvement in HIV-associated anemia and the potential mechanisms that might be involved included a reduction in opportunistic infections and the anemia of chronic disease, and an improvement in nutritional status. However, anemia in HIV-infected patients is also associated with zidovudine use [6], [7], [21]. Zidovudine is still recommended as part of the first-line regimens in resource-limited settings. The high prevalence of anemia that we found among newly diagnosed HIV-infected patients emphasizes the need for close monitoring of patients on a zidovudine-based ART regimen. We suggest that resource-limited countries should develop a plan to move towards tenofovir-based first-line regimens to optimize treatment options, and that continuing comparisons of hematological parameters among newly diagnosed HIV-infected patients before and after ART initiation are warranted.

Some limitations to our study should be noted. First, potential sample selection bias may have affected the findings. The HIV epidemic is serious in some areas and among some most-at-risk populations in China. The study population is not representative of the entire HIV-infected population in China and so the results may not be generalizable. Second, the design of the study was observational, we were able to examine potential associations but were unable to assess causation. Third, the data which allow the classification of the causes of anemia such as reticulocyte counts, erythropoietin levels, iron turnover, and parvovirus IgM titers were not measured. Fourth, the definition of anemia chosen for this study was different from the WHO criteria for the diagnosis of anemia. Our estimates of anemia prevalence may underestimate the true impact of anemia in this population. In addition, numerous parameters (environmental and nutritional factors, socio-economic status, lifestyle and others) that may affect haemoglobin levels [22] were not assessed in our study. Therefore, we were not able to determine the association between these factors and the prevalence of anemia. Furthermore, if these variables had been controlled for, some variables such as CD4 count might not have remained significant in the logistic regression model.

## Conclusions

Anemia is highly prevalent among adults with newly diagnosed HIV/AIDS in China. Older age, lower CD4 count and minority ethnicity are associated with an increased risk of anemia. It is important to routinely screen anemia for timely and adequate clinical management to reduce the morbidity of the patient. Further research is needed to evaluate the effects of treating anemia and the impact of anemia on ART and survival in HIV-infected patients.
